# Abnormal hemoglobin anti-Lepore Hong Kong compound with β^0^-thalassemia ameliorate thalassemia severity when co-inherited with α-thalassemia

**DOI:** 10.1038/s41598-024-56921-6

**Published:** 2024-03-20

**Authors:** Xiuqin Bao, Jicheng Wang, Danqing Qin, Cuize Yao, Jie Liang, Kailing Liang, Li Du

**Affiliations:** 1 Medical Genetic Center, Guangdong Women and Children Hospital, Xingnan Road 521, Guangzhou, Guangdong People’s Republic of China; 2 Maternal and Children Metabolic-Genetic Key Laboratory, Guangdong Women and Children Hospital, Guangzhou, 510010 Guangdong People’s Republic of China; 3 Thalassemia Diagnosis Center, Guangdong Women and Children Hospital, Guangzhou, 510010 Guangdong People’s Republic of China

**Keywords:** Anti-Lepore Hong Kong, Increased HbA2, Anti-Lepore Hong Kong compounded with β^0^-thalassemia and α-thalassemia, Single molecular real time sequencing, Genetics, Medical research

## Abstract

Abnormal hemoglobin anti-Lepore Hong Kong is a rare βδ fusion variants resulting from non-homologous crossover during meiosis. Anti-Lepore Hong Kong is known to consistently exhibit significantly increased level of HbA2. In this study, we used multiplex ligation-dependent probe amplification (MLPA) and single molecular real-time (SMRT) sequencing, as well as Sanger sequencing, to identify variants in five unrelated families with abnormal elevated HbA2 level. All probands in these five families were found to be heterozygous for anti-Lepore Hong Kong. Among them, two families showed co-occurrence of β^0^-thalassemia and α-thalassemia (–^SEA^/ or α^CS^α/). Heterozygotes for anti-Lepore Hong Kong displayed an average HbA2 level of 17.7% and behaved normal. However, when combined with β^0^-thalassemia and α-thalassemia, the probands exhibited higher HbA2 level (30.2–40.8%) and behaved with β-thalassemia trait. Furthermore, determination of the α/β-mRNA ratio revealed a slight downregulation of β-globin, similar to that of β-thalassemia minor. Our study is the first to identify compound heterozygotes for anti-Lepore Hong Kong, β^0^-thalassemia and α-thalassemia, provide valuable information for prenatal counseling.

## Introduction

Anti-Lepore hemoglobins (Hbs) are generated through non-equal crossover between β- and δ-globin genes during meiosis, resulting in hybrid βδ trains. Currently, nine types of anti-Lepore Hbs have been identified worldwide, including Hb Parchman (NG_000007.3: g.[63249_70661del;63571_70985dup])^1^, Hb Miyada (NG_000007.3: g.63249_70661dup)^2^, Hb P-India (NG_000007.3: g.63632_71046dup), Hb P-Congo (NG_000007.3: g/64557_ 71923dup)^3^, Hb Lincoln Park (HBD: c.412_414delGTG)^4^, Hb P-Nilotic (HbVar ID 748) ^5^, Hb anti-Lepore CHORI (NG_000007.3: g.63375_70786dup)^6^, Hb anti-Lepore Hong Kong (NG_000007.3: g.63154_70565dup)^7^ and Hb Palencia^1^ (http://globin.cse.psu.edu/). Most anti-Lepore Hbs do not significantly affect clinical presentation, and individuals who are heterozygous for these variants typically exhibit normal phenotypes. However, it has been reported that heterozygous of anti-Lepore Hong Kong compounded with β^0^-thalassemia will behave a mild thalassemia intermedia phenotype^7^. So et al.^8^ reported that a mild reduction of β-globin gene expression was seen in simple anti-Lepore Hong Kong heterozygotes when compared with β-thalassemia minor carriers (mean α/β ratio 3.62 vs. 17.23). However, anti-Lepore Hong Kong compounded with β^0^-thalassemia aggravated the imbalance of α/β when compared with β-thalassemia minor carriers (mean α/β ratio 25.59 vs. 17.23). These results may explain why the compound heterozygotes of anti-Lepore Hong Kong and β^0^-thalassemia behave a mild thalassemia intermedia phenotype. Consequently, it is of interest to investigate the effects of compounding anti-Lepore Hong Kong with both β^0^-thalassemia and α-thalassemia. In this study, we found two probands from five unrelated families who were compound heterozygotes for β^0^-thalassemia and α-thalassemia (–SEA/ or α^CS^α/). These two probands exhibited higher level of HbA2 (30.2%-40.8%) compared to the simple heterozygotes for anti-Lepore Hong Kong (17.7%) and behaved with β-thalassemia trait.

## Results

**Figure 1:**
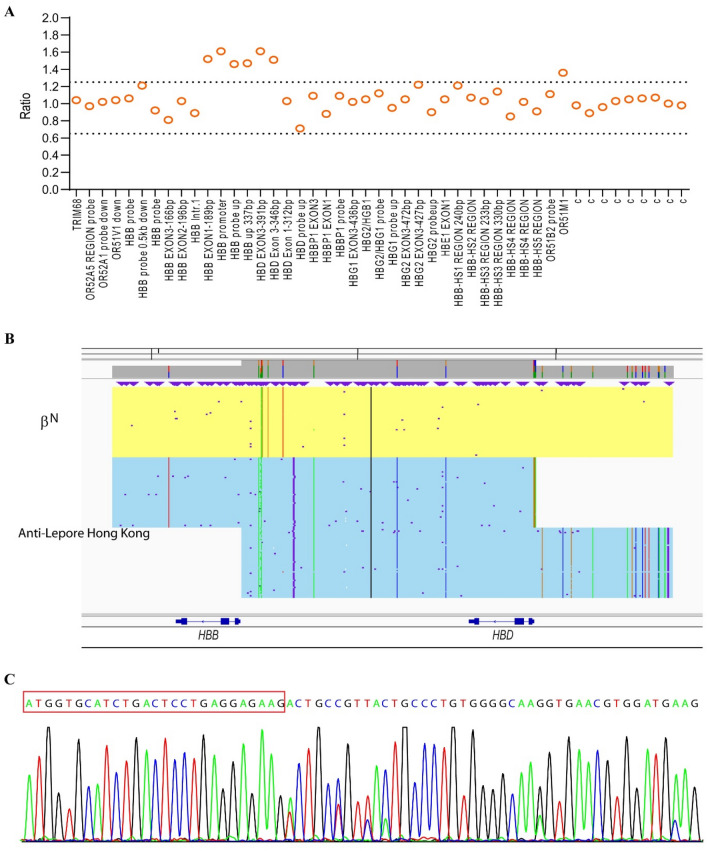
Genotyping of family A. (**A**) MLPA analysis in β-globin cluster. The red dashed line showed the ratio of 1.25, while the blue dash line showed the ratio of 0.65. (**B**) SMRT analysis in β-globin cluster. The light yellow and blue regions indicate the two alleles of β-globin gene cluster. The relative positions of the genes on chromosome 16 are indicated by blue boxes. The vertical colored lines indicate nucleotides A (green), T (red), C (blue) and G (orange) discordant with alignment to the hg38 reference sequence. (**C**) Sanger sequencing showed that the sequences of β- and δ- globin were the same from cap 22 to codon 8 in β-globin (the red box only showed codon 1–8).

Family A. The proband (AII1) was a 34-year-old woman from Guangdong province, China. She presented with hypochromic microcytic anemia, with Hb levels of 99 g/L, MCV of 75.8 fL, and MCH of 25.0 pg (Table 1). Hb quantification displayed an abnormal increase in the HbA2 + HbX zone (17.5%). Testing for common types of α- and β-thalassemia was negative. We then used MLPA and SMRT to determine the rare variants in β-globin cluster. We found a duplication spanning from the *HBD* exon 3 probe to the *HBB* exon 1 probe in MPLA (Fig. 1A). SMRT sequencing confirmed a duplication of approximately 7.4 kb in *HBB*-*HBD* region of chromosome 11 (hg38) from 5,227,105 to 5,234,516 (Fig. 1B). Sanger sequencing validated the SMRT sequencing result and confirmed the heterozygous genotype for anti-Lepore Hong Kong (Fig. 1C). The proband inherited the variant from her father (AI1), who was also a carrier of anti-Lepore Hong Kong (Table 1). Her 11-year-old son (AIII1) was also a heterozygote for anti-Lepore Hong Kong and exhibited a normal phenotype (Table 1).

**Table 1:** The phenotype and genotype data of the 5 families.

Family	Gender (M/F)	Age (years)	Hb (g/L)	RBC (10^12^/L)	HCT (%)	MCV (fL)	MCH (pg)	HbA (%)	HbA2 (%)	HbA2 + HbX (%)	HbA2 variant (%)	HbF (%)	Hb Bart’s (%)	Serum ferritin (ng/ml)	*HBA*	*HBB*
Family A																
AI1	M	59	148	4.80	44.3	92.4	30.9	81.6	0.0	18.0	0.4	0.0	0.0	210.82	αα/αα	Anti-Lepore Hong Kong/β^N^
AI2	F	62	126	4.04	37.8	93.4	31.2	97.5	2.5	0.0	0.0	0.0	0.0	143.94	NA	NA
AII1	F	34	99	3.97	30.1	75.8	25.0	82.1	0.0	17.5	0.4	0.0	0.0	8.34	αα/αα	Anti-Lepore Hong Kong/β^N^
AIII1	M	11	147	5.34	44.9	84.0	27.6	81.2	0.0	18.5	0.3	0.0	0.0	27.7	αα/αα	Anti-Lepore Hong Kong/β^N^
Family B																
BI1	M	34	127	6.77	42.8	63.2	18.8	95.0	5.0	0.0	0.0	0.0	0.0	NA	αα/αα	β^CD17^/β^N^
BI2	F	24	116	4.24	34.2	80.8	27.4	82.5	0.0	16.3	1.3	0.0	0.0	NA	α^CS^α/αα	Anti-Lepore Hong Kong/β^N^
BII1*	M	28^+^ w	130	3.76	39.6	105.2	34.6	2.2	0.0	0.4	0.0	95.2	2.2	NA	α^CS^α/αα	Anti-Lepore Hong Kong/β^CD17^
BII1	M	0 m	185	5.92	52.8	89.2	31.3	6.2	0.0	0.7	0.0	92.2	0.7	NA	α^CS^α/αα	Anti-Lepore Hong Kong/β^CD17^
BII1	M	6 m	92	5.27	29.2	55.4	17.5	43.2	0.0	27.4	0.0	29.4	0.0	87.5	α^CS^α/αα	Anti-Lepore Hong Kong/β^CD17^
BII1	M	10 m	94	5.34	27.8	52.1	17.6	NA	NA	NA	NA	NA	0.0	NA	α^CS^α/αα	Anti-Lepore Hong Kong/β^CD17^
BII1	M	13 m	NA	NA	NA	NA	NA	49.8	0.0	30.2	0.0	20.0	0.0	NA	α^CS^α/αα	Anti-Lepore Hong Kong/β^CD17^
BII1	M	18 m	103	5.69	32.0	56.3	18.2	51.3	0.0	32.8	0.0	15.9	0.0	27.45	α^CS^α/αα	Anti-Lepore Hong Kong/β^CD17^
BII1	M	24 m	103	5.75	31.9	55.5	17.9	NA	NA	NA	NA	NA	NA	NA	α^CS^α/αα	Anti-Lepore Hong Kong/β^CD17^
Family C																
CI1	F	25	107	4.96	31.4	63.3	21.6	53.4	0.0	40.8	3.4	0.4	0.0	59.24	–^SEA^/αα	Anti-Lepore Hong Kong/β^CD41–42^
CI2	M	25	129	6.26	44.5	71.0	20.6	96.3	2.4	0.0	0.0	1.3	0.0	NA	–^SEA^/αα	β^N^/β^N^
Family D																
DI1	F	26	113	5.56	35.4	63.3	20.3	97.6	2.4	0.0	0.0	0.0	0.0	NA	–^SEA^/αα	β^N^/β^N^
DI2	M	26	163	5.71	47.9	84.0	28.5	81.4	0.0	18.1	0.5	0.0	0.0	NA	αα/αα	Anti-Lepore Hong Kong/β^N^
Family E																
EI1	F	25	119	4.07	36.4	89.4	29.2	81.5	0.0	17.6	0.0	0.9	0.0	NA	αα/αα	Anti-Lepore Hong Kong/β^N^
EI2	M	NA	142	NA	NA	88.4	27.3	97.3	2.7	0.0	0.0	0.0	0.0	NA	αα/αα	β^N^/β^N^

F, Female; M, Male; NA, no available; 28^+^ w, 28 weeks of gestation; m, months.

*We followed this infant at 28 weeks of gestation until 24 months of age.

**Figure 2:**
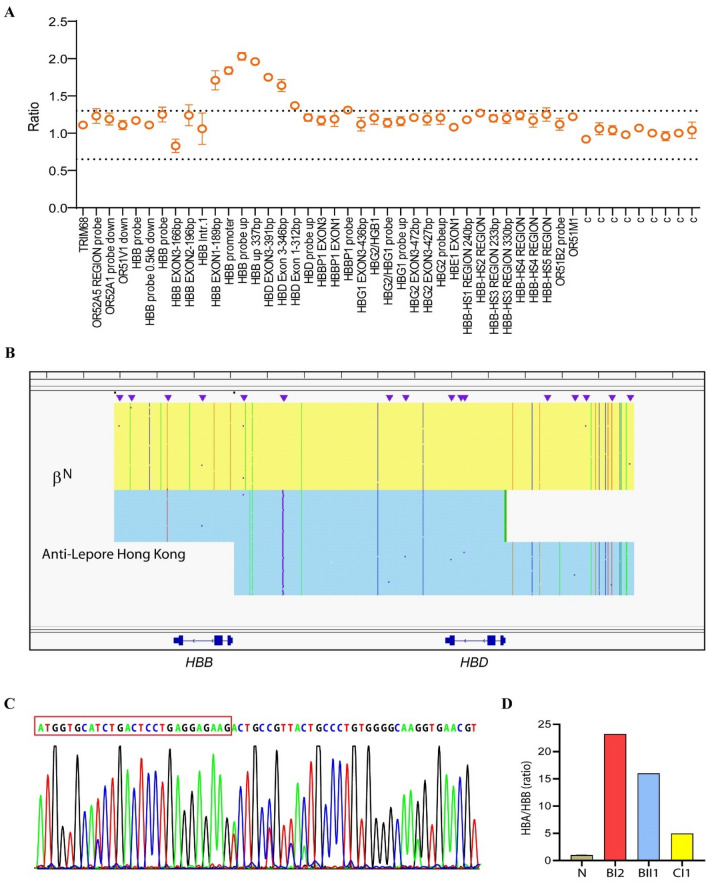
Genotyping of family B. (**A**) MLPA analysis in β-globin cluster. (**B**) SMRT analysis in β-globin cluster. (**C**) Sanger sequencing showed that the sequences of β- and δ- globin were the same from cap 22 to codon 8 in β-globin (the red box only showed codon 1–8). (**D**) Ratio of α- and β-mRNA in BI2 and BII1.

Family B. The proband (BI2) was a carrier of α^CS^α/, bahaving normal. However, Hb quantification displayed an increased level of HbA2 + HbX (16.3%) (Table 1). MLPA analysis showed a duplication spanning from the *HBD* exon 3 probe to the *HBB* exon 1 probe (Fig. 2A). SMRT sequencing confirmed the same duplication observed in family A (Fig. 2B). Sanger sequencing demostrated that the proband was a compound heterozygote for α^CS^α/αα and anti-Lepore Hong Kong (Fig. 2C). The proband’s husband (BI1) was found to be a heterozygote for β^CD17^/βN through detection of common types of thalassemia. Consequently, they sought prenatal diagnosis at our center. Genotyping results displayed that their son (BII1) inherited all the mutations from his parents, making him a compound heterozygote for α^CS^α/αα, anti-Lepore Hong Kong and β^CD17^/βN (Table 1). However, her son did no exhibited any manifestation at birth, and his hemotological parameters were normal (Table 1). Hb quantification showed an increase in HbA2 + HbX from 0.7 to 27.4% st 6 months old. Additionally, his Hb levels decreased from 185 to 92 g/L, along with a decrease in MCV (from 89.2fL at birth to 55.4fL at 6 months old) and MCH (from 31.3 pg at birth to 17.5 pg at 6 months old). At 18 months of age, his Hb level was 103 g/L, and the HbA2 + HbX level was 32.8%, slightly elevated compared to 27.4% at 6 months.

To assess the imbalance of α- and β-mRNA expression in BI2 and BII1, we performed quantitative real-time PCR and observed α- and β-mRNA ratios of 23.2 and 16.0, respectively, when normalized to the wild type ratio (Fig. 2D).

**Figure 3:**
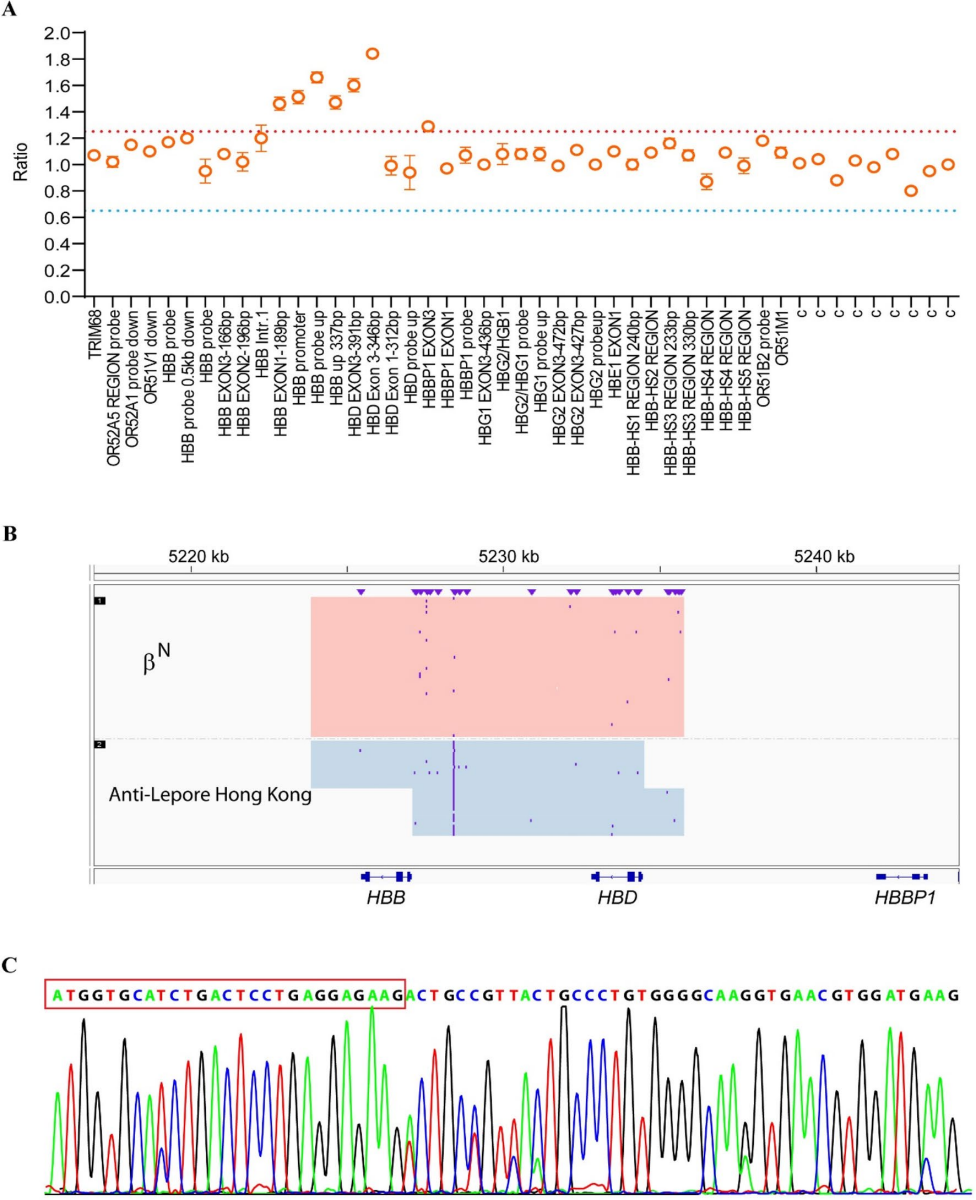
Genotyping of family C. (**A**) MLPA analysis in β-globin cluster. (**B**) SMRT analysis in β-globin cluster. The light pink and blue regions indicate the two alleles of β-globin gene cluster. (**C**) Sanger sequencing showed that the sequences of β- and δ-globin were the same from cap 22 to codon 8 in β-globin (the red box only showed codon 1–8).

Family C. The proband (CI1), a 25-year-old woman, presented with hypochromic microcytic anemia, with Hb levels of 107 g/L, MCV of 63.3 fL, and MCHof 21.6 pg. Hb analysis showed an abnormal increase in the HbA2 + HbX zone (40.8%, Table 1). Genetics analysis revealed the proband as a compound heterozygote for –^SEA^/αα and β^CD41–42^/β^N^. To further investigate rare variants, we performed MLPA and SMRT sequencing. We detected a fusion between the *HBB* and *HBD* gene (Fig. 3A), which apanned approximately 7.4 kb from 5,227,105 to 5,234,516 on chromosome 11 (hg38) (Fig. 3B). Sanger sequencing comfirned the SMRT sequencing result and showed that the proband also carried anti-Lopore Hong Kong, in addition to –^SEA^/αα and β^CD41–42^/β^N^ (Fig. 3C). Additionally, the ratio of α- and β-mRNA in the proband was 5.0 (Fig. 2D).

**Figure 4:**
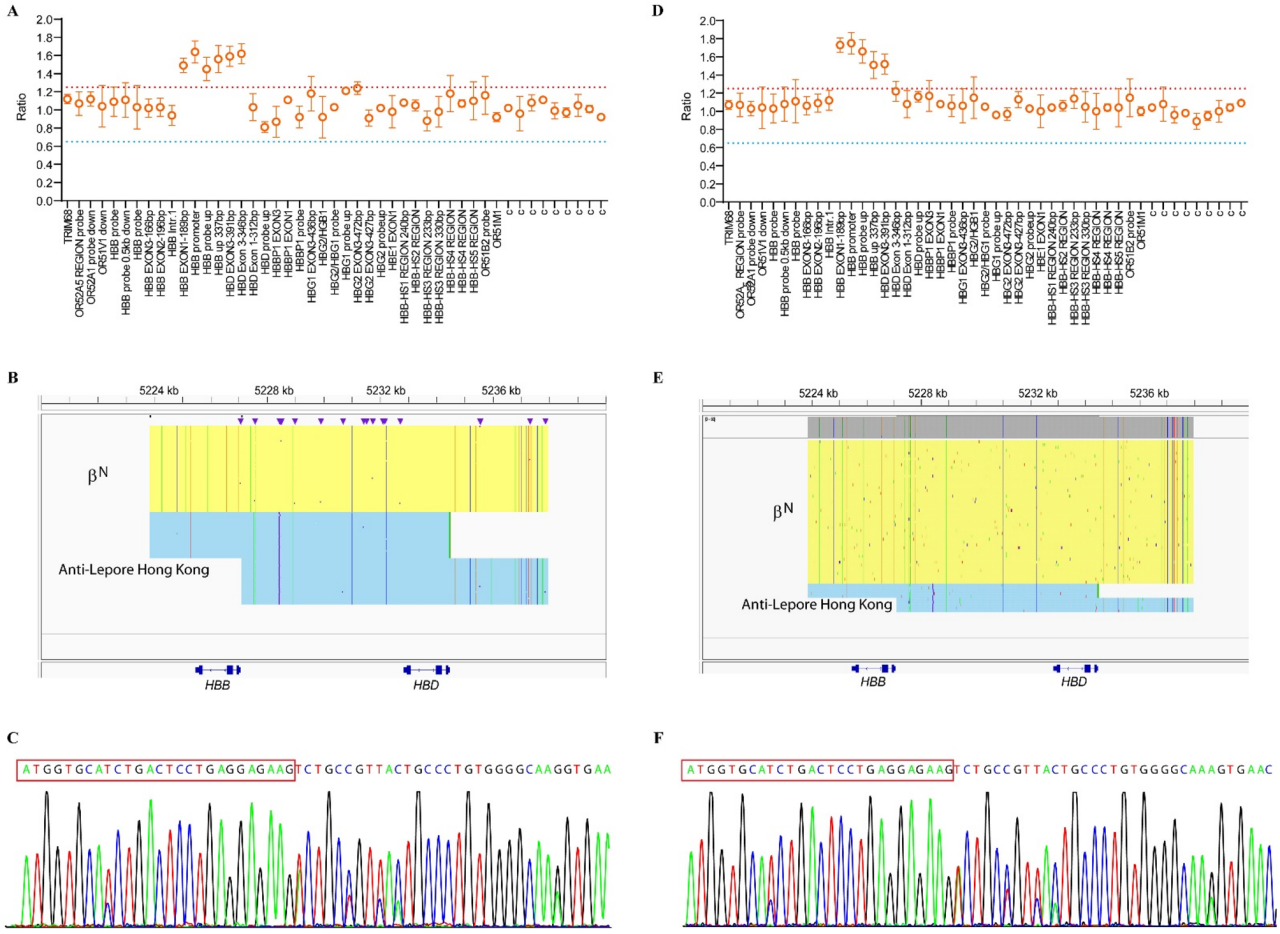
Genotyping of family D and E. (**A**–**C**) MLPA analysis in β-globin cluster (**A**), SMRT analysis in β-globin cluster (**B**) and Sanger sequencing (**C**) in family D. (**D**–**F**) MLPA analysis in β-globin cluster (**D**), SMRT analysis in β-globin cluster (**E**) and Sanger sequencing (**F**) in family E.

Family D and E. The probands in family D (DI2) and E (EI1) were both normal, despite exhibiting increased HbA2 + HbX levels (18.1% and 17.6% respectively) in Hb quantification analysis. Genetics analysis confirmed that they were carriers of anti-Lepore Hong Kong (Fig. 4**)**.

## Discussion

Anti-lepore Hong Kong was initially reported in 2006 by the university of Hong Kong^8^. The study identified two unrelated families and demonstrated that all heterozygotes for anti-Lepore Hong Kong behaved normal, which was consistent with the fonding of our study. The study also demostrated that anti-Lepore Hong Kong leads to overexpression of δ-globin chain and a mild thalassemia intermedia phenotype when co-inherited with β^0^-thalassemia. However, there were only one patient compounded with β^0^-thalassemia (β^CD41–42^/β^N^), which may not sufficient to establish the generalizability and accuracy of the findings. It is a pity that we did not identified any heterozygote for anti-Lepore Hong Kong compounded solely with β^0^-thalassemia in our study. Nonetheless, we identified two individuals who were heterozygotes for anti-Lepore Hong Kong compounded with α-thalassemia and β^0^-thalassemia. Patient 1 (BII1) from family B inherited the variants from his parents. We observed a gradual decrease in his Hb levels from 185 g/L at birth to 94 g/L at 10 months old, while the HbA2 + HbX level increased from 0.4 to 30.2%. These results suggest that the manifestation may be aggravated when anti-Lepore Hong Kong is compounded with α-thalassemia and β^0^-thalassemia. However, the other patient (CI1), a 25-year-old woman from family C, exhibited only thalassemia trait despite being a heterozygote for anti-Lepore Hong Kong, –^SEA^/αα and β^CD41–42^/β. Therefore, we suspected that the gradual decrease in Hb in BII1 may be a physiological response. Generally, after birth, as the baby establishes a spontaneous breathing system, the levels of hemoglobin tend to decrease. In addition, the ratio of α- and β-mRNA in these two patients were similar to that reported for β-thalassemia minor by So et al. ^8^. In addition, previous study showed that reduction of α-globin expression may provide an equally plausible approach to ameliorating clinically severe forms of β-thalassemia^9^. Hence, we supposed that heterozygotes for anti-Lepore Hong Kong compounded with α- and β^0^-thalassemia might alleviate the manifestation compared with the heterozygotes compound solely with β-thalassemia. The proband (AII1) in family A appears to have a mild thalassemia intermedia phenotype based on her hemotological parameters (Hb 99 g/L, MCV 75.8 fL, MCH 25.0 pg), and she is heterozygous of anti-Lepore Hong Kong. We speculated that the the mild thalassemia intermedia phenotype in AII1 may be due to physiological factors related to her pregnancy.

In conclusion, our study identified 5 unrelated families carrying anti-Lepore Hong Kong, and the heterozygotes in these families exhibited normal phenotypes, confirming previous fondings^8,10^. In addition, we identified two individuals who wer heterozygotes compounded with α- and β^0^-thalassemia, and they only displayed thalassemia traits, suggesting a milder manifestation compared to heterozygotes compounded solely with β-thalassemia. The study expands our understanding of the clinical phenotype of anti-Lepore Hong Kong when combined with common types of α- and β-thalassemia, providing valuable information in prenatal diagnosis (Supplementary information).

## Patients and methods

### Patients’ phenotype and common α- and β- thalassemia analysis

Family A. The proband (AII1) was a pregnant woman coming to our center for prenatal diagnosis due to hypochromic microcytic anemia. Hemoglobin analysis using Capillarys2 (Sejbia) showed an increased Hb A2 + Hb O (17.5%) (Table 1). Common types of α- [-α^3.7^ (rightward), -α^4.2^ (leftward), –^SEA^ (Southeast Asian), Hb Constant Spring (Hb CS or HBA2: c.427T>C), Hb Quong Sze (Hb QS or HBA2: c.377T>C) and Hb Westmead or HBA2: c.369C>G] and β-thalassemia [codons 41/42 (–TTCT) (HBB: c.126_127delCTTT), IVS-II-654 (C>T) (HBB: c.316-197C>T) –28 (A>G) (HBB: c.-78A>G), codons 71/72 (+ A) (HBB: c.216_217insA), codon 17 (AAG>TAG) (HBB: c.52A>T), codon 26 (GAG>AAG) (Hb E or HBB: c.79G>A), codon 31 (− C) (HBB: c.94delC), codons 27/28 (+ C) (HBB: c.84_85insC), IVS-I-1 (G>T) (HBB: c.92 + 1(G>T), codon 43 (GAG>TAG) (HBB: c.130G>T), − 32 (C>A) (HBB: c.-82>A), − 29 (A>G) (HBB: c.-79A>G), − 30 (T>C) (HBB: c.-80T>C), codons 14/15 (+ G) (HBB: c.45_46insG), Cap + 40–43 (–AAACA) (HBB: c.-11_-8delAAACA), initiation codon (ATG>AGG) (HBB: c.2 T>G) and IVS-I-5 (G>C) (HBB: c.92 + 5G>C)] were detected using suspension array system. Her father and son, behaving normal, also had an abnormal elevated HbA2 + Hb O (18.0% and 18.5% respectively). Her mother was normal. All of them were negative in the detection of common types of α- and β-thalassemia. The data were showed in Table 1.

Family B. The proband (BI2) was referred for prenatal diagnosis because of the abnormal increased Hb A2 + Hb O (16.3%). The detection of common types of α- and β-thalassemia displayed that she was α^CS^α/αα, while her husband was β^CD17^/heterozygotes, who behaved hypochromic microcytic anemia. Her son was inherited the both two mutations from father (βCD17) and mother (αCSα). In addition, her son also had a progressive increased Hb A2 + Hb O level (increased from 0.4% in core blood in 28 weeks of gestation to 30.2% in peripheral blood (PB) in 13 months old) (Table 1).

Family C. A 25-year-old woman (CI1) was referred for genetic analysis due to hypochromic microcytic anemia and abnormal increased Hb A2 + Hb O (40.8%). α- and β-thalassemia analysis showed that she was compound heterozygote of –^SEA^ and β^CD41–42^. Her husband was heterozygote of –^SEA^ behaving thalassemia trait (Table 1).

Family D and family E. The probands in family D (DI2) and family E (EI1) both had raised HbA2 (18.1% and 17.6% respectively). However, they all behaved normal (Table 1).

### Hematological analysis

Peripheral blood (PB) sample was collected, and hematological parameters were analyzed by using a Sysmex XN5000 automated hematology analyzer (Sysmex Corporation, Kobe, Japan). Hb quantification was performed by automated capillary electrophoresis system (CE) (Sebia Capillarys 2, France), which has high sensitivity.

### Rare genotype analysis

Multiplex ligation-dependent probe amplification (MLPA) was used to detect the rearrangements of β-globin gene cluster (SALSA MLPA KIT P102 D1 HBB, MRC‐Holland, Amsterdam, the Netherlands) according to the manufacturer’s instructions. Ratio > 1.25 was defined as duplication. Sanger sequencing was used to screen the rare mutations in α- and β-globin gene.

#### Single molecular real-time (SMRT) sequencing

We used SMRT sequencing to precise determine the rearrangement of β-globin gene cluster. Experiments were conducted by Berry Genomic Corporation (Beijing, China) as described in previous study^9^. Briefly, genomic DNA was subjected to PCR with primers covering the majority of known structural variations, SNVs and indels in HBA1, HBA2 and HBB regions, as well as the DNA region encompassing the duplication probes by MLPA. PCR products were ligated with barcoded adaptors by a one-step end-repair and ligation reaction to construct pre-libraries, which were pooled together by equal mass and converted to single-molecule real-time dumbbell (SMRTbell) library by Sequel Binding and Internal Ctrl Kit 3.0 (Pacific Biosciences). Then, SMRTbell library was sequenced under circular consensus sequencing (CCS) mode on Sequel II platform (Pacific Biosciences). The converted CCS reads were aligned to hg38 and the precise regions of duplication were determined.

### Quantative real-time PCR (qRT-PCR)

We used qRT-PCR to determine the imbalance of α- and β-mRNA expression. Total RNA extracted from GPA + cells using TRIzol reagent (Life Technologies) was reverse-transcribed into cDNA using a PrimeScript RT Reagent Kit with gDNA Eraser (Takara, China). A comparative qPCR assay with SYBR green dye-containing SuperArray PCR master mix (Takara) was performed on an ABI Prism 7500 system (Life Technologies) with ACTB as a reference gene. The primers were used as follow: α-globin, F: 5ʹ-CACGCTGGCGAGTATGGT-3ʹ, R: 5ʹ-GCGGGAAGTAGGTCTTGGT-3ʹ; β-globin, F: 5ʹ-GGTGAACGTGGATGAAGTT-3ʹ, R: 5ʹ-CCTCTGGGTCCAAGGGTAG-3ʹ.

### Ethics approval and consent to participate

All the procedures performed in studies involving human participants were in accordance with the ethical standards of the institutional and national research committee and with the 1964 Helsinki Declaration and its later amendments or comparable ethical standards. Informed consent was obtained from all individual participants included in this study. Approval for this study was obtained as outlined by the protocol #202101025 approved by the Medical Ethics Committee of Guangdong Women and Children Hospital.

### Supplementary Information

Supplementary Information.

## Data Availability

All the data and materials were available in this study. The DNA sequencing data were available with the accession number PRJNA1081482 in the National Center for Biotechnology Information Bioproject database. Additional information is also available upon reasonable request to the corresponding authors.
